# Oral Care and Quality Evaluation: A Multicentric Study on Periodontal Treatment

**DOI:** 10.3290/j.ohpd.a44444

**Published:** 2020-07-04

**Authors:** Filippo Graziani, Luigi Minenna, Dimitra Karapets, David Herrera, Marco Nisi, Stefano Gennai, Mario Gabriele, Nicola Discepoli, Morena Petrini, Urška Marhl, Marina Perić, Laurence Adriaen, Bettina Alonso, Philippe Bouchard, Daniele Cardaropoli, Raffaele Cavalcanti, Tali Chackartchi, Fernando Franch-Chillida, Rodolfo Gianserra, Adrian Guerrero, Luca Landi, Silvia Masiero, Magda Mensi, Paolo Moratti, Francesco Oreglia, Antonio Rupe, Ignazio Sanz Sanchez, Nicola Marco Sforza, Diego Capri, Ion Zabalegui, Mariano Sanz, Maurizio Tonetti, Cristiano Tomasi

**Affiliations:** a Full Professor, Department of Periodontology, School of Dentistry, University of Pisa, Italy. Hypothesis, experimental design production, treatment, data collection, study design, manuscript production.; b Clinical Tutor, School of Dentistry, University of Ferrara, Italy. Treatment, data collection.; c Private Dental Practicioner, Bologna, Italy. Manuscript production.; d Full Professor and Associate Dean, School of Dentistry, Complutense University of Madrid, Spain; Director of ETEP (Etiology and Therapy of Periodontal Diseases) Research Group, Complutense University Madrid. Treatment, data collection.; e PhD Student, Department of Surgical, Medical and Molecular Pathology, School of Dentistry, University of Pisa, Italy. Statistical analysis, manuscript production.; f Former PhD Student, Department of Surgical, Medical and Molecular Pathology, School of Dentistry, University of Pisa, Italy. Statistical analysis, manuscript production.; g Full Professor of Oral Surgery and Dean, School of Dentistry, University of Pisa, Italy. Treatment, data collection.; h Assistant Professor, Department of Periodontology, School of Dentistry, University of Pisa, Italy, and School of Dentistry, University of Siena, Italy. Treatment, data collection.; i Research Fellow, School of Dentistry, University of Pisa; Clinical Assistant, Unit of Dentistry and Oral Surgery, University Hospital, Pisa, Italy. Manuscript production.; j PhD Student, Department of Surgical, Medical and Molecular Pathology, School of Dentistry, University of Pisa, Italy. Manuscript production.; k PhD Student, Department of Surgical, Medical and Molecular Pathology, School of Dentistry, University of Pisa, Italy. Manuscript production.; l Private Periodontal Practitioner, Palma de Mallorca, Spain. Treatment, data collection.; m Assistant Professor, School of Dentistry, Complutense University of Madrid, Spain; ETEP (Etiology and Therapy of Periodontal Diseases) Research Group, Complutense University, Madrid. Treatment, data collection.; n Full Professor of Periodontology, U.F.R. d’odontologie, Université Paris Diderot, France; Head, Oral Pathology – Oral Surgery and Periodontal Surgery Clinical Unit, Hôpital Rothschild AP-HP, France. Treatment, data collection.; o Private Dental Practitioner, Turin, Italy. Treatment, data collection.; p Private Dental Practitioner, Bari, Italy. Treatment, data collection.; q Periodontologist, Department of Periodontology, Hadassah and Hebrew University, Faculty of Dental Medicine, Jerusalem, Israel. Treatment, data collection.; r Private Periodontal Practitioner, Palma de Mallorca, Spain. Treatment, data collection.; s Private Periodontal Practitioner, Rome, Italy. Treatment, data collection.; t Private Periodontal Practitioner, Rome Marbella, Spain. Treatment, data collection.; u Private Periodontal Practitioner, Rome, Italy. Treatment, data collection.; v Private Dental Practitioner, Saronno, Italy. Treatment, data collection.; w Assistant Professor, Department of Periodontology, School of Dentistry, University of Brescia, Italy. Treatment, data collection.; x Paolo Moratti Private Dental Practitioner, Reggio Emilia, Italy. Treatment, data collection.; y Francesco Oreglia Private Periodontal Practitioner, Verona, Italy. Treatment, data collection.; z Antonio Rupe Private Dental Practitioner, Benevento, Italy. Treatment, data collection.; * Assistant Professor, Complutense University of Madrid, Spain; ETEP (Etiology and Therapy of Periodontal Diseases) Research Group, Complutense University Madrid; Private Periodontal Practitioner, Madrid. Treatment, data collection.; # Private Dental Practitioner, Bologna, Italy. Treatment, data collection.; § Private Dental Practitioner, Bologna, Italy. Treatment, data collection.; ** Private Periodontal Practitioner, Bilbao, Spain. Treatment, data collection.; ## Full Professor, Department of Periodontology, School of Dentistry, Complutense University of Madrid, Spain; Director, ETEP (Etiology and Therapy of Periodontal Diseases) Research Group, Complutense University Madrid. Treatment, data collection, study design, manuscript production.; §§ Clinical Professor, Department of Periodontology, University of Hong Kong. Treatment, data collection, study design, manuscript production.; + Associate Professor, Department of Periodontology, Institute of Odontology, The Sahlgrenska Academy, University of Gothenburg, Sweden. Treatment, data collection, statistical analysis.

**Keywords:** oral health related quality of life, periodontitis, quality of care

## Abstract

**Purpose:**

No information is available on the perception of the quality of care in patients treated for periodontitis. The purpose of this article was to assess how periodontitis-affected patients perceive the quality of periodontal treatment (PT) and to measure the factors which may influence it.

**Materials and Methods:**

306 subjects who completed PT were invited to participate. Questionnaires and visual analogic scales (VAS) evaluating perception of quality of care, symptoms, and oral health related quality of life (OHRQoL) were handed out. Oral and periodontal indicators were collected before and after treatment. The impact of different factors on perception of quality was assessed with a regression model.

**Results:**

Quality evaluation was high yet unrelated for both patients and clinicians (p = 0.983). Quality was negatively influenced by the number of residual oral infections (p < 0.001), patient’s age (p = 0.07) and presence of residual pain at completion of PT (p = 0.02). Professionalism, kindness of the staff and communication skills were the characteristics mostly appreciated. The OHRQoL was influenced by the number of residual teeth (p < 0.001), increasing age of patients (p = 0.08), number of residual infections (p < 0.01) and pain (p = 0.04).

**Conclusions:**

Patients’ quality perception appeared to be influenced by clinical and emotional aspects. Oral care providers should be aware of the impact of non-clinical factors in patients’ appreciation of quality of treatment.

The outcome of periodontal treatment has been routinely and historically evaluated through variation of both biological and clinical surrogate parameters of periodontal health.^29^ Nevertheless, patients’ opinions and perception may differ from these traditional clinical end points.^24^ Thus, surrogate measures have been increasingly complemented by so-called patient-based outcomes or patient-reported outcomes, i.e. subjective measures which precisely capture patients’ perspectives of the disease and/or therapy with the aim of being tangible to the patient.^15,31^

Patients’ appreciation and feelings about the disease and the treatment are important, as patients constitute the key element within the overall treatment.^28^ Patient compliance is another important element in successful periodontal treatment, but this is related to the ability of the patients to understand their role in maintaining therapy results and their adherence to the provided instructions.^19,26^

Compliance is influenced by many factors, and the perception of the quality of care received is one of the most relevant. Patients convinced by the quality of the care provided are more likely to comply with treatment,^13^ take an active role in treatment^10^ and continue using medical care services offered by a given a health provider once the active therapy has been completed.^20^ Moreover, quality assessment may also enhance patient comprehension of the treatment plan and foster the interpersonal relationship.^4^

In spite of these clear advantages, quality evaluation during and after periodontal treatment has not been assessed in the scientific literature. Thus, the aim of the present study was to assess the quality of the treatment as perceived by periodontal patients and to measure the factors which may influence such perception.

## Materials and Methods

### Study Design

This was a multicentre study based on questionnaires, visual analogic scales (VAS) and clinical data of patients previously treated for periodontitis. The protocol of the study received approval from the leading ethics committee (protocol # 2668/2016). The population of this study consisted of patients diagnosed with periodontitis who during the previous 6 months had undergone nonsurgical and surgical periodontal therapy in periodontal specialist clinics in Europe, and were selected by phone call. Questionnaires and forms were previously translated into English, Spanish, Italian, and French according to region, and were e-mailed to selected periodontal specialist clinics.

### Quality Evaluation

In one of the first supportive periodontal treatment appointments, patients were handed a questionnaire comprising questions and a number of different VAS. Patients filled out the questionnaires in a separate room in the absence of any member of the dental team. On a 10-cm VAS, patients were asked to rate the overall quality of care received. The scale ranged from ‘no quality at all (completely inadequate)’ to ‘highest possible level of quality (completely adequate)’.

### Patient Factors

Questionnaires also contained a section on symptoms: based on a Likert scale (no – always – often – sometimes – rarely), patients were asked to report whether they had experienced any pain or sensitivity to cold/hot substances during treatment. They were also asked to indicate if pain and sensitivity were still present on a VAS (0 = ‘no pain/sensitivity’ to 10 = ‘unbearable pain/sensitivity’). Aesthetics were also evaluated with a VAS ranging from 0 (worsening), 5 (no changes) to 10 (improvement). A Likert scale was also used to indicate whether gingival bleeding was still present.

Patients were also asked to choose one or more of the characteristics they liked and appreciated the most about their therapist among dental team-related issues (technical professionalism, ability to explain, punctuality, kindness of the staff, cost effectiveness)^6,9,25^ and practice-related issues (website and IT facilities, practice layout, technology, overall hygiene and cleanliness).^22^

Finally, the oral-health related quality of life (OHRQoL) was evaluated with the Oral Health Impact Profile-14 (OHIP-14) questionnaire.^30^

### Clinician Factors

Clinicians were required to fill in a form providing information concerning their age, academic studies, teaching and research activity, and working environment. Furthermore, they had to fill in a second form containing information relevant to the patient’s age, gender, systemic health status and smoking habits before and after treatment as well as the type of periodontal therapy (non-surgical, surgical or both), the total number of clinical sessions and patient’s total cost for treatment. Furthermore, the therapists provided clinical periodontal parameters of the treated patients pre- and post-treatment, that is, full-mouth plaque score (FMPS) in categories (< 25%, 25–50%, > 50%), number of teeth and number of sites with periodontal pocket depth (PPD) < 4 mm and PPD ≥4 mm, and bleeding on probing (BOP). PPD was measured with a periodontal probe as the distance between the gingival margin and the deepest part of the gingival pocket. Information concerning the average full-mouth bone resorption, in terms of average tooth length, was also collected and expressed as an overall evaluation on peri-apical radiographs. Finally, the therapists had to assess whether the treatment goals had been achieved and to evaluate the overall quality of the treatment with the same VAS scale used for the patient’s quality evaluation.

### Statistical Analysis

Data were entered into an Excel 2003 (Microsoft; Redmond, WA, USA) database and proofread for entry errors. The database was subsequently locked, imported into statistical software for the analysis (SPSS 21, IBM, USA). Frequency distribution, mean values and standard deviation (SD) of all parameters were calculated for descriptive purposes.

The primary outcome was the patient’s quality evaluation on the VAS scale. The secondary outcomes were the clinician’s quality evaluation and the patient’s OHRQoL.

For the analysis of OHIP-14, additional parameters, such as prevalence, extent, and severity, were calculated.^31^ Briefly, prevalence was defined as the proportion of participants who reported one or more items as ‘fairly often’ or ‘very often’; extent was defined as the number of items reported as ‘fairly often’ or ‘very often’; and severity was the simple sum of the response codes for the 14 items, with 56 being the highest score with the highest impact on quality of life.^31^

To evaluate the impact of the different tested factors on the primary and secondary outcomes, a multilevel regression model was constructed using specially designed software (MLWin 2.32, Centre for Multilevel Modelling, Bristol University, UK), considering the operator as the highest level and the patient as the lowest. After having run an empty model, the predictors, i.e. all measured variables, were inserted one by one and tested for significance by the use of a Wald test. All significant predictors were inserted in the final model and the model improvement was evaluated using of a chi-squared test on the -2log-likelyhood decrease.

The following parameters were investigated for association with the evaluated outcomes:

- At the patient level: gender, age, presence of a systemic disease, smoking habit (yes/no/former), non-surgical approach (staged/full-mouth), surgery (yes/no), number of clinical sessions, number of teeth present, number of residual pockets with PPD ≥ 5 mm after completion of the treatment, number of residual BOP-positive sites after completion of the treatment, plaque score post treatment, pain during treatment, presence of residual pain after treatment, sensitivity change, presence of residual sensitivity, perception of aesthetic variations and gum bleeding.
- At the clinician level: country, presence of a specialist degree (yes/no), age, years of professional practice and number of dental chairs.

## Results

### Study Population

Demographic, clinical and medical characteristics of the study population are given in Table 1. 306 patients in total agreed to participate and filled out the questionnaires from October 2013 to June 2015 given them by 18 periodontists who worked in periodontal specialist clinics in various European countries. Seventeen patients (SD 12.9) on average were treated by each clinical centre. Clinical centres were mainly located in southern Europe, namely Italy, Spain, France and Israel (11 centres from Italy, 5 from Spain, 1 from France and 1 from Israel). The clinicians involved had been practicing dentistry for an average of 21.5 years (SD 7.1) and 10 were also specialists in periodontology.

**Table 1:** Demographic characteristics of the study sample

Variable	Number
Patients	306
Age (SD, range)	51.7 (11.5, 24 – 92)
Gender F/M (%)	175 / 131 (57.2%/42.8%)
Smokers (yes/no/former) (%)	212 / 47 / 47 (70%/15%/15%)
Cigarettes/day in smokers: mean (SD)	14.6 (6.1)
Quit smoking for treatment: n (percentage)	15 (7%)
Systemic disease (% of diseased)	43 (14%)
Diabetes (% of diseased)	12 (28%)
Hypertension (% of diseased)	16 (37%)
Number of teeth at initial exam (SD)	25.7 (3.7)
Number of clinical sessions (SD)	5.8 (4.7)

SD: standard deviation; F: female; M: male.

Participants involved in the study were primarily females (57%) and current smokers (69%). The sample included mostly systemically healthy participants. Among those with systemic diseases (14% of interviewed participants), type 2 diabetes and hypertension were most frequently reported.

On average, at initial examination, participants presented more than 25 teeth (SD 3.7), while the average number of clinical sessions was approximately 6 (SD 4.7). All patients underwent non-surgical treatment. Fifty-five patients received full-mouth treatment, whereas the remainder received conventional/quadrant treatment. One hundred fifty-seven patients were also surgically treated.

### Treatment Performance on Clinical Periodontal Parameters

As reported in Table 2, plaque control improved as the percentage of patients with FMPS < 25% increased from 7% to 83% of the sample (p < 0.01). Participants with FMPS above 50% decreased from 62% to 2% (p < 0.01). BOP decreased from an average of 48.5% (SD 29.7) to an average of 8.8% (SD 9.3) (p < 0.01) and sites with PPD ≥ 5 mm decreased from an average of 25.2% (SD 18.9) to an average of 5.2% (SD 6.9) (p < 0.01).

**Table 2:** Periodontal parameters at baseline and after treatment

Parameter	Baseline	Final
Number of teeth, mean (SD)	25.7 (3.7)	25.1 (3.8)
FMPS <25%, 25-50%, >50%	7%, 31%, 62%	86%, 12%, 2%
BOP+ No. sites, mean (SD)	69.4 (44.9)	12.2 (13.3)
BOP+ %, mean (SD)	48.5% (29.7)	8.8 % (9.3)
No. of pockets ≥ 5 mm mean (SD)	36.8 (28.1)	7.5 (10.1)
% Pockets ≥ 5 mm mean (SD)	25.2 % (18.9)	5.2 % (6.9)

SD: standard deviation; FMPS: full-mouth plaque score; BOP+: positive for bleeding on probing.

### Patients’ Quality Evaluation of the Treatment and Reported Outcomes

The quality evaluation score was calculated on 281 questionnaires. The overall quality evaluation score was very high, with an average VAS score of 9.33 (SD 1.4). The quality score was negatively influenced by the number of residual pockets of PPD ≥ 5 mm, the age of the patient (although not statistically significant) and the presence of residual pain at the end of the treatment (Table 3). The variance at the clinician level was not statistically different from 0, indicating no significant difference in terms of average quality perception between patients treated in different centres.

**Table 3:** Multilevel model (2 levels, clinician and patient) with quality evaluated by patients on a VAS scale as the dependent variable

Response: quality patient	Empty		Final		p
Fixed	Parameter	SE	Parameter	SE	
Intercept	9.362	0.099	9.347	0.081	<0.001
Age			-0.012	0.007	0.07X
No. sites PD ≥5 mm			-0.026	0.008	0.001
Residual pain			-0.094	0.041	0.02X
Random					
Level: clinician					
Variance	0.056	0.056	0.009	0.035	0.80X
Level: patient					
Variance	1.833	0.152	1.757	0.147	<0.001
-2*log likelihood:	1060.982		1021.938		<0.001

R^2^ = 0.07; VAS: visual analogic scale; SE: standard error; PD: probing depth.

Cost-effectiveness was evaluated as more than satisfactory (8.9, SD 1.7) and, in general, symptoms were significantly reduced at the final re-evaluation, as residual sensitivity, pain and bleeding were mostly not reported (Appendix). The most highly appreciated characteristics were professionalism (reported by 93.1% of the patients), kindness of the staff (74.8%) and communication skills (54.9%). On the other hand, the lowest scores were given for costs (9.8%), website (6.9%) and practice structural facilities (4.9%). Cleanliness, technological resources and punctuality were moderately appreciatied (rated by 23.5%, 18.3% and 13.1%, respectively, of the patients as most appreciated characteristics). Upon completion of treatment, the OHRQoL, as measured through OHIP-14, indicated an extent of 0.8 (SD 1.7) and a severity of 9.8 (SD 9.2) indicating minimal, if any, impact of oral conditions on the overall perceived quality of life (Table 4). Of the various areas of OHRQoL, psychological discomfort was the one that received the highest values, as 17.6% of the sample still felt self-conscious about their oral conditions at the end of the treatment. The multilevel regression model indicated that the higher the number of teeth and age of the participants, the lower is the impact of oral condition perceived quality of life (Table 5). Conversely, the number of residual pockets, the presence of residual pain or pain during treatment had a negative impact on the quality of life. No effect on quality evaluation was noted according to patient’s gender, type of treatment (surgical vs non-surgical) and clinical centre.

**Table 4:** OHIP-14 at completion of periodontal therapy

OHIP subscale and item	% Blank/don’t know	Prevalence: % subjects reporting at least 1 item fairly often or very often	Extent: no. of items rated fairly often or very often	Severity: mean score (SD)
Functional limitation				
Had trouble pronouncing words	22.2	2.9	9	0.4 (0.9)
Felt that sense of taste had worsened	22.9	2.0	6	0.4 (0.8)
Physical pain				
Had painful aching in mouth	12.7	6.5	20	1.0 (1.1)
Was uncomfortable eating	12.7	11.1	34	1.2 (1.2)
Psychological discomfort				
Has been feeling self- conscious	11.4	17.6	54	1.4 (1.2)
Has felt tense	15.4	13.4	41	1.3 (1.2)
Physical disability				
Diet has been unsatisfactory	19.6	3.9	12	0.6 (0.9)
Has had to interrupt meals	18.0	1.0	3	0.5 (0.7)
Psychological disability				
Finds it difficult to relax	18.0	5.2	16	0.8 (1.0)
Has been a bit embarrassed	18.3	8.8	27	0.9 (1.1)
Social disability				
Has been irritable with other people	20.9	2.0	6	0.6 (0.9)
Has had difficulty doing usual jobs	21.9	0.3	0	0.4 (0.7)
Handicap				
Has found life less satisfying	22.5	3.3	10	0.6 (0.9)
Has been totally unable to function	23.9	1.0	3	0.2 (0.6)
Total	18.6 (4.2)	30.4	0.8 (1.7)	9.8 (9.2)

**Table 5:** Multilevel model (2 levels, clinician and patient) with total OHIP-14 as evaluated by patient as dependent variable

Response: TotOHIP14	Empty	SE	Final	SE	p
Fixed					
Intercept	9.925	1.108	11.725	1.18	<0.001
Age			-0.125	0.053	0.017
Smoker			2.872	1.644	0.08XX
Former smoker			0.41	1.868	0.8XX
N teeth present			-0.574	0.167	<0.001
N sites PPD ≥5mm			0.155	0.059	0.009
Residual pain			0.566	0.28	0.04X
Pain during treatment			1.799	0.569	0.002
Random					
Level: operator					
Variance	10.845	6.416	6.543	4.395	0.14X
Level: patient					
Variance	73.822	7.416	60.888	6.186	<0.001
-2*log likelihood:	1528.528		1450.122		

R^2^ = 0.07; VAS: visual analogic scale; SE: standard error; PD: probing depth.

**Table 6:** Multilevel model (2 levels, clinician and patient) with quality as evaluated from clinician as dependent variable

Response: Quality Op.	Empty	SE	Final	SE	p
Fixed					
Intercept	8.806	0.196	8.774	0.182	<0.001
Patient age			-0.014	0.006	0.01X
Smoker			-0.367	0.179	0.04X
Former smoker			0.042	0.181	0.81X
Full-mouth			0.371	0.179	0.04X
N sites PPD ≥5mm			-0.017	0.008	0.02X
N sites BOP+			-0.017	0.006	0.004
Random					
Level: clinician					
Variance	0.588	0.227	0.339	0.147	0.02X
Level: patient					
Variance	1.124	0.094	0.989	0.086	<0.001
-2*log likelihood:	941.852		814.585		<0.001

R^2^ = 0.07; VAS: visual analogic scale; SE: standard error; PD: probing depth; BOP+: positive for bleeding on probing.

### Clinicians’ Quality Evaluation of the Treatment

Quality analysis of the therapists was analysed on 278 questionnaires. Clinicians defined treatment as of high quality, as the average VAS value was 8.8 (SD 1.3) out of 10. Clinicians’ quality evaluation was not related to the quality perceived by the patients (Pearson correlation 0.001, p = 0.983).

Quality evaluation was negatively influenced by the number of residual pockets of PPD ≥ 5 mm, number of bleeding sites and patient smoking habits (Table 5). Moreover, a centre-effect was noted, indicating differences among centres in terms of quality evaluation. Patient’s age was significantly inversely related to the quality evaluation. On the other hand, the type of non-surgical treatment delivery, specifically, the full-mouth approach was associated with a higher quality evaluation by the clinicians.

## Discussion

This study evaluated the perception of quality of periodontal treatment upon its completion and assessed the factors that may influence it from the patients’ perspective. Our findings suggest that the perceived quality is high and influenced by the presence of residual disease and persistence of symptoms such as pain/discomfort at the end of the treatment. Clinicians’ quality evaluation was also high, but interestingly, it differed from that of the patients and was influenced by different factors.

Quality of care/treatment can be defined as ‘the degree to which health services for individuals and populations increase the likelihood of desired health outcomes and are consistent with current professional knowledge’.^17^ Understanding the impact of quality is crucial in contemporary medicine. The assessment of quality goes beyond mere technicalities, as an efficient use of resources and proper risk management complement professional performance and especially the patient’s satisfaction.^33^

Given that there were no validated questionnaires measuring the quality of care in periodontology, a new tool – a VAS scale assessing the quality of treatment – was designed for this study. Results show that patients perceived and evaluated the overall quality of treatment as high. The sample in our study judged the treatment received as completely meeting their expectations. However, their perception of the quality was negatively influenced by the presence of residual pockets and lingering pain.

The fact that residual pocketing has an influence on the perception of quality might imply that the patient subjectively perceives the presence of disease irrespective of the symptoms (inflammatory indices such as BOP did not have an impact). Nevertheless, it is unlikely that presence of pockets has an impact, since it is considered a clinical surrogate endpoint, objectively intangible to patients.^15^ It could be speculated that the presence of the disease might affect the quality of life, which could then further influence their perception of the quality of treatment. This was in fact observed in the multilevel model, indicating that the OHIP-14 score depends on the number of residual pockets. Moreover, the reader should bear in mind that clinicians were not prevented from communicating the findings to their patients. It is thus possible that the patients might have been influenced by the therapist’s report.

The impact of residual pain after treatment on the quality evaluation seems easier to understand. Post-operative pain and its management in hospital settings can be clearly associated with perceived quality of care and patients’ experience, as patients with higher levels of reported post-surgical pain evaluated the quality of care lower.^16^ The absence of pain after non-surgical treatment has positive impacts for non-surgical periodontal therapy on OHRQoL scores.^2,23,34^

Professionalism, kindness of the staff and communication skills were characteristics most highly appreciated by patients in this study; all three were reported by more than half of the participants. These findings emphasise the great importance of emotional care and compassion in the dentist-patient relationship. Empathetic communication in the patient-therapist rapport may reduce anxiety and fear.^1,18,32^ Indeed, positive suggestions and the ability to correctly inform the patient may lower the patients’ levels of pain.^21^

On the other hand, the characteristics that proved nearly irrelevant to the patients were costs, website and practice structural facilities. Interestingly, studies on patients’ perception of health care prices and their association with quality of care show varying results. Patients’ perception of dental service suppliers showed that the more expensive ones were perceived as more competent and thus patients were more willing to purchase their dental services.^9^ Nevertheless, more recent studies showed that higher prices are perceived as synonymous with higher quality by a substantial minority of the population.^5,14,27^ Further research on the influence treatment cost has on the perceived quality of care in dentistry should be encouraged. Practice websites do not seem to be relevant for the patients. A study conducted on dental advertising services showed a significant discrepancy between the rating of importance of websites between dentist and patients, indicating that for the latter only a minority of them consider it a relevant tool.^7^

Interestingly, quality evaluation by the therapists was also high, despite being influenced by different factors, and as such their evaluation was not related to that of the patients. Treatment was correctly performed as seen by the observed changes of parameters.^3,8^ Clinicians’ quality of care perception was inversely related to the number of residual pockets of PPD ≥ 5 mm, number of bleeding sites and patient smoking habits, all surrogate clinical endpoints which can be objectively assessed by the therapist. The type of delivery was also important, as shown by the full-mouth approach being associated with a higher quality evaluation score by clinicians, even though there is no clear clinical evidence that the full-mouth approach is more effective than a staged-treatment approach.^11,12^

The authors are aware of the intrinsic limitations and strengths of the study. Participants enrolled in this study were selected through a phone call, and not randomly selected. Furthermore, the number of enrolled participants was not consistent among the centres. Clinicians could not be blinded, as they had to provide relevant patient relevant information, periodontal parameters and evaluate the overall quality of the treatment they had performed. Nevertheless, this is a study with an important sample on a novel topic, given that so far no studies on the evaluation of quality of care in periodontology have been conducted. Large prospective studies are needed to capture important differences among subjects with different levels of quality appreciation.

## Conclusions

Appreciation of quality of care does not appear to be correlated between patients and clinicians. Presence of residual pockets showed an impact on both patients and clinicians. Nevertheless, patients were greatly influenced by emotional aspects, such as the presence of residual pain, and appreciated characteristics related to soft communicative skills.

### Implications

Clinicians should be aware of the importance of non-clinical factors in organising and providing high quality care to patients. Acknowledging factors that influence patients’ perception of quality of treatment may actively contribute to improving clinical effectiveness and patient compliance. Prospective studies evaluating other aspects of the quality evaluation should be encouraged in order to provide insights and improve quality of care in dentistry, enhancing effectiveness and compliance.

## Appendix Patient-reported outcomes

**Table:**

Question	VAS mean (SD)				
Do you still feel pain?	1.0 (1.9)				
Do you still have sensitivity?	2.6 (2.8)				
Have you noticed changes in aesthetics?	6.2 (3.1)				
	Likert scale				
	No	Rarely	Sometimes	Often	Always
Did you feel pain during treatment?	44.8%	19.9%	27.5%	6.5%	1.3%
Have you noticed any sensitivity change?	34.0%	20.6%	27.8%	13.0%	4.6%
Did your gums bleed very recently?	67.3%	20.3%	10.6%	1.0%	1.0%

## References

[ref1] Armfield J, Heaton L (2013). Management of fear and anxiety in the dental clinic: a review. Aust Dent J.

[ref2] Åslund M, Suvan J, Moles DR, D’Aiuto F, Tonetti MS (2008). Effects of two different methods of non-surgical periodontal therapy on patient perception of pain and quality of life: a randomized controlled clinical trial. J Periodontol.

[ref3] Badersten A, Nilvéus R, Egelberg J (1981). Effect of nonsurgical periodontal therapy. I. Moderately advanced periodontitis. J Clin Periodontol.

[ref4] Baker KA (1995). The role of dental professionals and the patient in plaque control. Periodontol 2000.

[ref5] Carman KL, Maurer M, Yegian JM, Dardess P, McGee J, Evers M (2010). Evidence that consumers are skeptical about evidence-based health care. Health Aff.

[ref6] Chakraborty G, Gaeth GJ, Cunningham M (1993). Understanding consumers’ preferences for dental service. J Health Care Mark.

[ref7] Clow KE, Stevens RE, McConkey CW, Loudon DL (2007). Attitudes of dentists and dental patients toward advertising. Health Mark Q.

[ref8] Cobb CM (2002). Clinical significance of non-surgical periodontal therapy: an evidence-based perspective of scaling and root planing. J Clin Periodontol.

[ref9] Crane FG (1996). The relative effect of price and personal referral cues on consumers’ perception of dental services. Health Mark Q.

[ref10] Donabedian A (1988). The quality of care. How can it be assessed?. JAMA.

[ref11] Eberhard J, Jervøe-Storm P-M, Needleman I, Worthington H, Jepsen S (2008). Full-mouth treatment concepts for chronic periodontitis: a systematic review. J Clin Periodontol.

[ref12] Eberhard J, Jepsen S, Storm P-M, Needleman I, Worthington H V (2015). Full-mouth treatment modalities (within 24 hours) for chronic periodontitis in adults. Cochrane Database Syst Rev.

[ref13] Guldvog B (1999). Can patient satisfaction improve health among patients with angina pectoris?. Int J Qual Health Care J Int Soc Qual Health Care.

[ref14] Hibbard JH, Greene J, Sofaer S, Firminger K, Hirsh J (2012). An experiment shows that a well-designed report on costs and quality can help consumers choose high-value health care. Health Aff.

[ref15] Hujoel PP (2004). Endpoints in periodontal trials: the need for an evidence-based research approach. Periodontol 2000.

[ref16] Idvall E, Hamrin E, Sjöström B, Unosson M (2002). Patient and nurse assessment of quality of care in postoperative pain management. Qual Saf Health Care.

[ref17] Institute of Medicine (US) Committee to Design a Strategy for Quality Review and Assurance in Medicare (1990). Medicare: A Strategy for Quality Assurance: Volume 1.

[ref18] Kloostra PW, Eber RM, Inglehart MR (2007). Anxiety, stress, depression, and patients’ responses to periodontal treatment: periodontists’ knowledge and professional behavior. J Periodontol.

[ref19] Lee CT, Huang HY, Sun TC, Karimbux N (2015). Impact of patient compliance on tooth loss during supportive periodontal therapy. J Dent Res.

[ref20] Marquis MS, Davies AR, Ware JE (1983). Patient satisfaction and change in medical care provider: a longitudinal study. Med Care.

[ref21] Mistiaen P, van Osch M, van Vliet L, Howick J, Bishop FL, Di Blasi Z (2016). The effect of patient-practitioner communication on pain: a systematic review. Eur J Pain.

[ref22] Moser HR, Freeman GL (2017). An empirical analysis of the public’s attitudes toward advertising chiropractic services: A comparative cross-sectional study. J Med Mark Device, Diagnostic Pharm Mark.

[ref23] Needleman I, McGrath C, Floyd P, Biddle A (2004). Impact of oral health on the life quality of periodontal patients. J Clin Periodontol.

[ref24] Ng SKS, Leung WK (2006). Oral health-related quality of life and periodontal status. Community Dent Oral Epidemiol.

[ref25] Niamtu J (2008). Marketing the oral and maxillofacial surgery practice through positive employee relations. Oral Maxillofac Surg Clin North Am.

[ref26] Pachêco-Pereira C, Spivakovsky S (2016). Patient compliance and periodontal outcomes. Evid Based Dent.

[ref27] Phillips KA, Schleifer D, Hagelskamp C (2016). Most Americans do not believe that there is an association between health care prices and quality of care. Health Aff (Millwood).

[ref28] Ramseier CA, Suvan JE (2010). Health behavior change in the dental practice.

[ref29] Savage A, Eaton KA, Moles DR, Needleman I (2009). A systematic review of definitions of periodontitis and methods that have been used to identify this disease. J Clin Periodontol.

[ref30] Slade GD (1997). Measuring oral health and quality of life. Dent Ecol.

[ref31] Tsakos G, Allen PF, Steele JG, Locker D (2012). Interpreting oral health-related quality of life data. Community Dent Oral Epidemiol.

[ref32] Vatne JF, Gjermo P, Sandvik L, Preus HR (2015). Patients’ perception of own efforts versus clinically observed outcomes of non-surgical periodontal therapy in a Norwegian population: an observational study. BMC Oral Health.

[ref33] WHO Working Group on the Principles of Quality Assurance, World Health Organization. Regional Office for Europe (1985). The principles of quality assurance : report on a WHO meeting, Barcelona, 17-19 May 1983.

[ref34] Wong RMS, Ng SKS, Corbet EF, Keung Leung W (2012). Non-surgical periodontal therapy improves oral health-related quality of life. J Clin Periodontol.

